# Pregnancy disrupts the accuracy of automated fT4 immunoassays

**DOI:** 10.1530/ETJ-22-0145

**Published:** 2022-10-11

**Authors:** Heleen I Jansen, Antonius E van Herwaarden, Henk J Huijgen, Rebecca C Painter, Jacquelien J Hillebrand, Anita Boelen, Annemieke C Heijboer

**Affiliations:** 1 Department of Clinical Chemistry, Endocrine Laboratory, Amsterdam UMC location Vrije Universiteit Amsterdam, Amsterdam, The Netherlands; 2 Amsterdam Gastroenterology, Endocrinology & Metabolism, Amsterdam, The Netherlands; 3 Department of Clinical Chemistry, Endocrine Laboratory, Amsterdam UMC location University of Amsterdam, Amsterdam, The Netherlands; 4 Radboud University Medical Center, Department of Laboratory Medicine, Nijmegen, The Netherlands; 5 Department of Clinical Chemistry, Red Cross Hospital, Beverwijk, The Netherlands; 6 Department of Obstetrics and Gynaecology, Amsterdam UMC Vrije Universiteit Amsterdam, Amsterdam, The Netherlands; 7 Amsterdam Reproduction & Development Research Institute, Amsterdam, The Netherlands

**Keywords:** pregnancy, free thyroxine, immunoassay, LC-MS/MS

## Abstract

**Objective:**

Thyroid hormone measurements are often performed in pregnant women, as hypo- and hyperthyroidism during pregnancy can severely affect the fetus. Serum free thyroxine (fT4) measurements are well known for their analytical challenges, due to low serum concentrations and the subtle equilibrium between free and bound T4 (to thyroid-binding globulin (TBG), transthyretin and albumin). Pregnant women have high TBG concentrations due to an increase in human chorionic gonadotropin (hCG) and estrogen and lower albumin concentrations which change the equilibrium and may affect the validity of fT4 measurements in their samples. As accurate serum fT4 measurements in pregnant women are important for the long-term health of the fetus, we aimed to evaluate the accuracy of several fT4 immunoassays in the serum of pregnant women.

**Methods:**

FT4 was measured in healthy controls and pregnant women using a candidate-reference method (LC-MS/MS) and five commercially available automated immunoassays (Alinity (Abbott), Atellica (Siemens), Cobas (Roche), Lumipulse (Fujirebio) and UniCel DXI (Beckman Coulter)). Method comparisons (Bland Altman plots and Passing and Bablok analyses) were performed.

**Results:**

Serum samples from both healthy controls (*n*  = 30) and pregnant women (*n*  = 30; mean gestational age, 24.8 weeks) were collected. The fT4 immunoassays deviated +7 to +29% more from the LC-MS/MS in serum samples of pregnant women than healthy controls (falsely high).

**Conclusions:**

Our results indicate that immunoassays overestimate fT4 in pregnant women, which might lead to an overestimation of thyroid status. Physicians and laboratory specialists should be aware of this phenomenon to avoid drawing false conclusions about thyroid function in pregnant women.

## Introduction

Thyroid hormone measurements are often performed in pregnant women, since undiagnosed or inadequate treatment of thyroid disorders is associated with adverse perinatal and neonatal outcomes (1, 2, 3). Untreated maternal hypothyroidism may lead to long-term consequences for the fetus, including impaired psychomotor development, neuropsychological development and memory function (4, 5, 6). Maternal hypothyroidism can especially affect fetal brain development in early pregnancy, since the fetus does not produce thyroid hormone itself until 16–20 weeks (7). Untreated maternal hyperthyroidism can not only have fetal consequences such as intra-uterine growth restriction but also life-threatening maternal consequences as thyroid storm and congestive heart failure (8). Serum thyroid-stimulating hormone (TSH) and free thyroxine (fT4) are measured to diagnose these thyroid disorders.

Thyroxine (T4) of 99.95% is bound to serum proteins of which 75% binds to thyroid-binding globulin (TBG), 10% to transthyretin and 12% to albumin, while only 0.02% is circulating unbound fT4 (9, 10). Serum fT4 is commonly measured using automated immunoassays (IAs) and this measurement is notorious for its analytical challenges because of low concentrations in the picomolar range and the subtle equilibrium between free and bound T4. Importantly, this equilibrium must not be disturbed in the measurement procedure. Sparse literature shows that pregnancy increases serum TBG concentrations which cause increased T4-binding capacity and may compromise the validity of the fT4 measurement method compared to serum of healthy controls (11, 12). Furthermore, decreased albumin concentrations during pregnancy seem to influence the accuracy of fT4 radioimmunoassays (RIAs) (13, 14), although a causal relationship has never been established. Moreover, literature focusing on the influence of automated fT4 IA accuracy is still lacking. The accuracy of the IA measurement of hormones such as vitamin D, testosterone and cortisol in pregnancy is shown to be affected by increased binding globulins (15, 16, 17, 18). Although these hormones are measured as total hormone concentration, we speculated that altered concentrations of binding globulins could influence the measurement of fT4 as well. However, the extent to which pregnancy along with increased TBG or decreased albumin concentrations affects the accuracy of various fT4 IAs has not yet been fully elucidated. Furthermore, the conventional reference measurement procedure of fT4 using liquid chromatography tandem-mass spectrometry (LC-MS/MS), preceded by an equilibrium dialysis method, is aimed to minimally disturb the physiological equilibrium between fT4 and bound T4 in serum to ensure an accurate measurement of fT4. This method measures fT4 directly, whereas IAs measure fT4 in an indirect manner. The LC-MS/MS method was created according to prevailing conventions and can be used as a reference method to measure fT4 (19, 20).

This study aimed to perform a method comparison between several commercially available automated fT4 immunoassays and an fT4 candidate-reference method (equilibrium dialysis combined with LC-MS/MS) in pregnant women and healthy controls to establish the potential bias between current clinically used fT4 immunoassays and an LC-MS/MS reference method in serum samples of pregnant women.

## Materials and methods

### Samples

Serum samples were obtained from 30 pregnant women (mean age, 33.4 years) and 30 healthy controls (mean age, 39.5 years) (11 men, 19 women) in March, April and May 2021 after written informed consent was given. The local Medical Ethical Committee of the Amsterdam UMC, location Academic Medical Centre confirmed that ethical approval was not required since this research was set up for laboratory analysis quality improvement. Additional blood samples from pregnant women were collected during the first blood withdrawal of an oral glucose tolerance test at the outpatient clinic of Amsterdam UMC location AMC (The Netherlands). Healthy controls were recruited among employees at Amsterdam UMC. Pregnant women were excluded if they used thyroid medication (e.g. levothyroxine, thiamazol); healthy controls were excluded if they used thyroid medication or took oral contraceptives. Gestational age at inclusion ranged from 14 to 32 weeks (mean, 24.8 weeks). In pregnant women, blood was sampled between 08:00 and 10:00 h while fasting, whereas blood was sampled between 08:00 and 17:00 h in healthy controls and was not routinely taken in fasted state. All samples were handled identically; the samples were aliquoted after centrifugation (5 min at 1900 ***g***) and kept frozen at −20°C until analysis. Storage time did not exceed 7 months. Before analyses, all samples were thawed, vortexed and centrifuged after which batch analysis took place.

### Methods

#### Immunoassays

Serum fT4 concentrations were measured using five different commercially available automated competitive immunoassays: Alinity (Abbott), Atellica (Siemens), Cobas (Roche; Gen III), Lumipulse (Fuijrebio) and UniCel DXI (Beckman Coulter). Two of these assays were one-step immunoassays (Atellica and Lumipulse) and three of these assays were two-step immunoassays (Alinity, Cobas and Unicel DXI). Albumin concentrations were measured using the Cobas (Roche) colorimetric assay (bromocresol purple method). Serum TBG concentrations were measured using an RIA (Thermo Fischer Scientific). Analyses using Alinity, Atellica, Cobas, Lumipulse and RIA were performed in batch at the Endocrine Laboratory of Amsterdam UMC. Analyses using UniCel DXI were performed in batch at the laboratory of Red Cross Hospital (RKZ) Beverwijk.

#### LC-MS/MS

Serum fT4 concentrations were measured at the department of Laboratory Medicine at Radboud UMC Nijmegen according to the method described by Jansen *et al.* (2022; in preparation). In short, conventional equilibrium dialysis (ED) in serum was performed according to the defined conventions (21). These conventions required a biochemical composition of dialysis buffer resembling the ionic environment of serum during dialysis, namely pH of 7.4 ± 0.03 (at 37°C), a temperature of 37°C ± 0.5°C and required the use of two compartments of serum and dialysis buffer separated by a regenerated cellulose membrane containing an identical volume. This resulted in a physically separated fraction of T4 that was followed by a liquid–liquid extraction and subsequently measured using isotope-diluted (ID) LC-MS/MS. Certified primary reference material was used as calibrator (IRMM-468; VWR International, NL) and all volumetric steps were gravimetrically performed. All measurements were performed in triplicate. Imprecision was calculated and showed a total CV of 3.9% at 13.1 pmol/L and 3.1% at 32.0 pmol/L.

### Statistics

All serum fT4 concentrations in pregnant women and healthy controls, measured using the five different immunoassays, were compared to the concentrations measured using the LC-MS/MS method. Parametric tests were used when the outcomes were normally distributed. Independent *t* tests were performed to assess the difference in mean fT4 (for all IAs and the LC-MS/MS method) and TBG concentrations between pregnant women and healthy controls. Albumin concentrations were not normally distributed, so a Mann–Whitney *U* test was performed to assess the difference in median albumin concentrations between pregnant women and healthy controls. Passing and Bablok regression analyses, Bland–Altman plots and calculated Pearson correlation coefficients were used for method comparisons. To provide better insight into the absolute effect of the matrix of pregnant women, we recalculated the IA results toward the LC-MS/MS results based on the method comparison for healthy controls. The fT4 concentrations of pregnant women were recalculated by the following formula: (initial fT4 measured using IA – intercept)/slope. Intercept and slope were derived from the Passing and Bablok regression analyses between the respective IA and LC-MS/MS fT4 results in healthy controls. The recalculated concentrations from pregnant women were used to make Bland–Altman plots. All statistical analyses were performed using Medcalc (version 18.5, Medcalc Software). *P* ≤ 0.05 was considered statistically significant.

## Results

**Figure 1:**
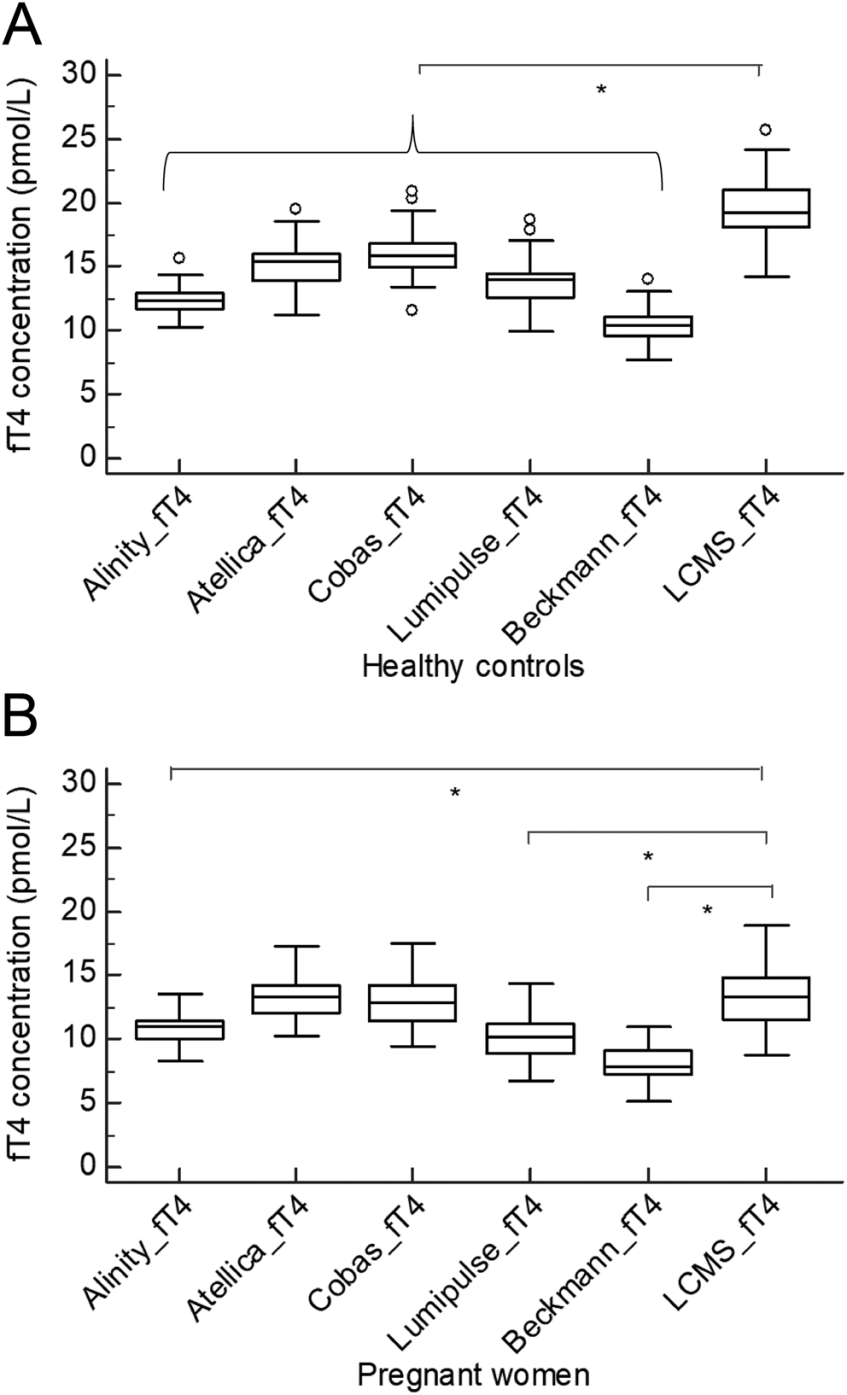
Box-and-whisker plots of the fT4 concentrations (x-axis) in both healthy controls (A) and pregnant women (B) using five different immunoassays and an LC-MS/MS method (y-axis). ^*^ *P* < 0.05.

**Figure 2:**
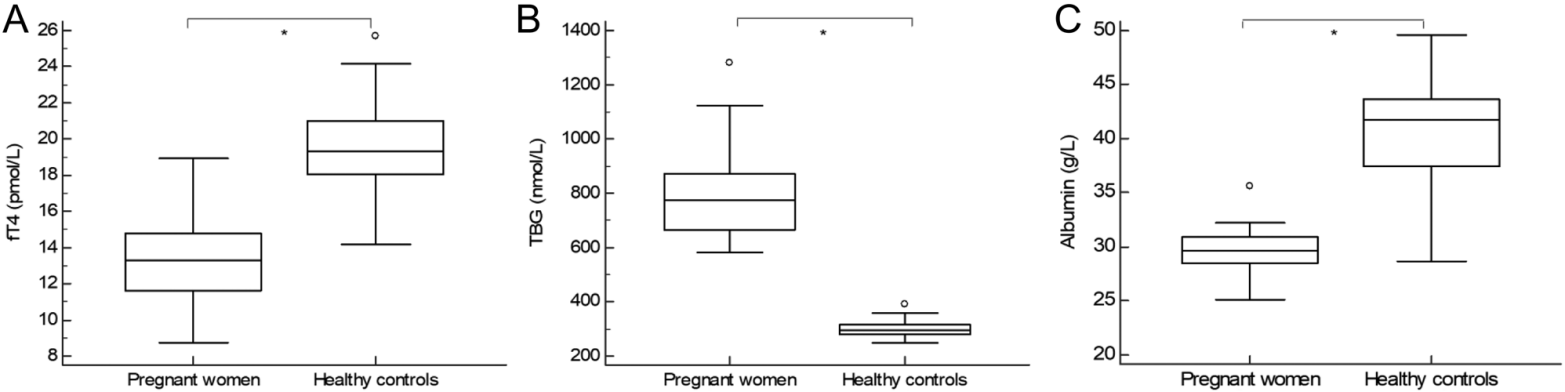
Box-and-whisker plots of the fT4 (A), TBG (B) and albumin concentrations (C) in pregnant women (*n*  = 30) and healthy controls (*n*  = 30). fT4 concentrations measured using LC-MS/MS. ^*^ *P*  < 0,0001.

Figure 1 shows that fT4 concentrations in healthy controls were significantly lower when measured using all tested IAs compared to the LC-MS/MS (percentage difference Alinity −36%, Atellica −23%, Cobas −19%, Lumipulse −29% and UniCel DXI −46%). In the pregnant population, this was also the case for the Alinity, Lumipulse and UniCel DXI IA (percentage difference respectively −19, −24 and −39%), whereas the Atellica and Cobas IAs did not measure significantly lower compared to the LC-MS/MS (percentage difference respectively +0.7 and −2%). Figure 2 shows that fT4 concentrations were significantly lower in pregnant women compared to healthy controls (mean pregnant women LC-MS/MS 13.2 pmol/L; 95% CI: 12.3–14.1 pmol/L; mean healthy controls LC-MS/MS 19.6 pmol/L; 95% CI: 18.7–20.5 pmol/L; *P* < 0.0001; data IAs not shown). TBG concentrations were significantly higher in pregnant women compared to healthy controls (mean pregnant women 802 nmol/L; 95% CI: 736–868 nmol/L; mean healthy controls 302 nmol/L; 95% CI: 290–313 nmol/L; *P* < 0.0001), whereas albumin concentrations were significantly lower in pregnant women compared to healthy controls (median pregnant women 29.7 g/L; IQR: 28.5–30.9 g/L; median healthy controls 41.8 g/L; IQR: 37.4–43.6 g/L; *P* < 0.0001).

**Figure 3:**
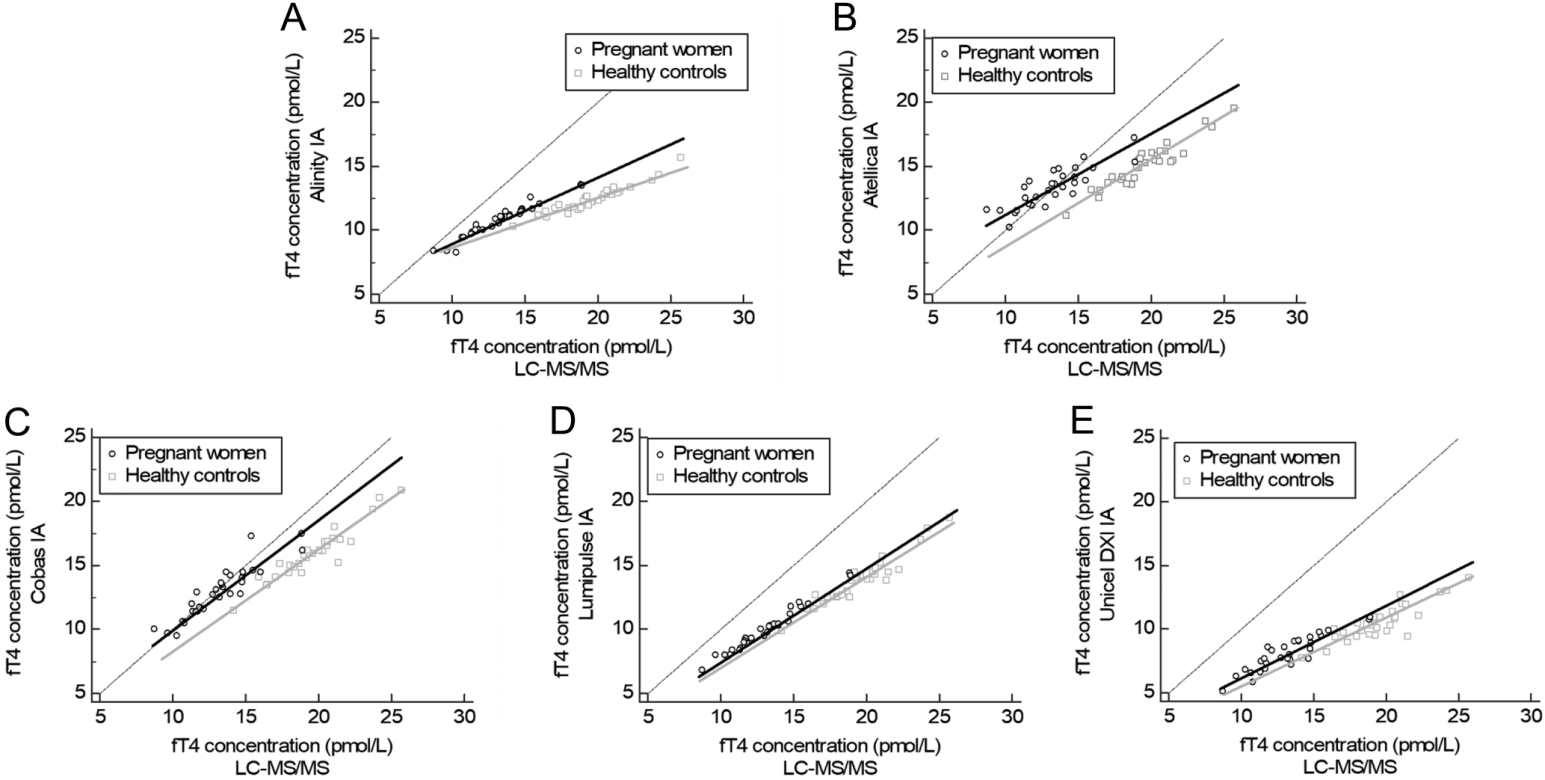
Passing and Bablok regression analyses for the five automated immunoassays in pregnant women and healthy controls. On the x-axis, the fT4 concentrations were measured using LC-MS/MS and, on the y-axis, the fT4 concentrations using the respective immunoassays are shown. (A) Alinity; (B) Atellica; (C) Cobas; (D) Lumipulse; (E) UniCel DXI.

**Figure 4:**
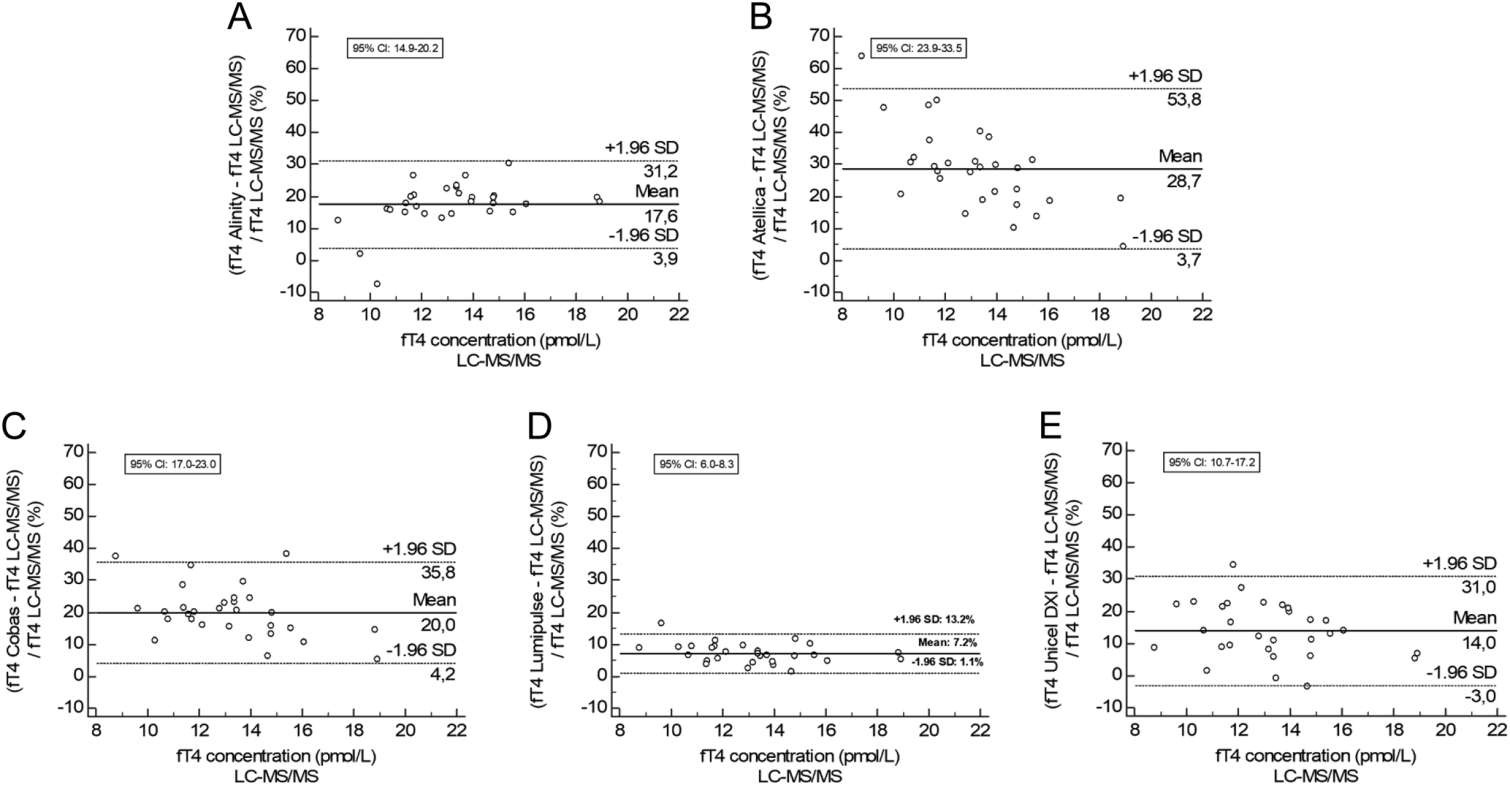
Bland–Altman plots for the five automated immunoassays in pregnant women compared to healthy controls. To provide better insight into the absolute effect of the matrix of pregnant women, we recalculated the IA results toward the LC-MS/MS results based on the method comparison for healthy controls by the following formula: (initial fT4 measured using IA – intercept)/slope. The recalculated concentrations from pregnant women were used to make these Bland–Altman plots. On the x-axis, the mean fT4 concentrations in pregnant women measured using LC-MS/MS were shown and, on the y-axis, the % deviation of the respective IAs compared to the LC-MS/MS assay were shown. (A) Alinity; (B) Atellica; (C) Cobas; (D) Lumipulse; (E) UniCel DXI.

Table 1 shows the slope and intercept results from the Passing and Bablok regression analyses of the five automated IAs compared to the LC-MS/MS and their correlation coefficients. Figure 3 presents the Passing and Bablok regressions of the five automated IAs compared to the LC-MS/MS in both pregnant women and healthy controls. This figure shows that, for all IAs, the regression line of the samples from pregnant women exceeds the regression line of the samples from the healthy controls, indicating a positive deviation of fT4 using the IA in samples from pregnant women after controlling for the deviation between the IA and LC-MS/MS in healthy controls. To quantify this positive deviation, recalculated fT4 concentrations from pregnant women, as described in the method section, were used to make Bland–Altman plots and are shown in Fig. 4. All IAs showed an increased deviation in fT4 level in pregnant women compared to healthy controls; in other words, all IAs produce falsely high results in samples from pregnant women. The deviation varied from 7.2% (1.1–13.2%) for the Lumipulse IA up to 28.7% (3.7–53.8%) for the Atellica IA (Fig. 4) and was statistically significant for all IAs.

**Table 1:** Slope, intercept and correlation coefficient according to Passing and Bablok regression for each fT4 assay compared to the LC-MS/MS method in healthy controls and pregnant women.

Assay compared to LC-MS/MS	*n*	Passing and Bablok regression			95% CI	r
		Slope	95% CI	Intercept (pmol/L)		
Alinity						
Healthy controls	30	0.41	0.36 to 0.48	4.37	3.05 to 5.43	0.9576
Pregnant women	30	0.53	0.49 to 0.60	3.78	2.89 to 4.31	0.9734
Atellica						
Healthy controls	30	0.69	0.61 to 0.78	1.65	−0.07 to 3.33	0.9493
Pregnant women	30	0.65	0.50 to 0.85	4.67	2.23 to 6.64	0.8524
Cobas						
Healthy controls	30	0.79	0.70 to 0.85	0.49	−0.69 to 2.22	0.9475
Pregnant women	30	0.84	0.72 to 1.01	1.63	−0.35 to 3.24	0.9193
Lumipulse						
Healthy controls	30	0.71	0.63 to 0.80	−0.004	−1.71 to 1.66	0.9515
Pregnant women	30	0.73	0.69 to 0.78	0.40	−0.28 to 1.00	0.9890
Unicel DXI						
Healthy controls	30	0.55	0.44 to 0.66	−0.13	−2.20 to 1.94	0.8732
Pregnant women	30	0.58	0.47 to 0.68	0.47	−0.95 to 1.95	0.9093

## Discussion

In this study, we performed a method comparison between five automated commercially available fT4 IAs routinely used in clinical laboratories and an fT4 (candidate) reference method (equilibrium dialysis combined with LC-MS/MS) in pregnant women and healthy controls to evaluate whether fT4 IAs measure accurately in serum of pregnant women. Our results indicated that fT4 concentrations in pregnant women measured using IAs were often falsely increased leading to a potential overestimation of thyroid hormone status. Results will be discussed below.

We showed that fT4 concentrations measured using both LC-MS/MS and IAs were significantly lower in the samples of pregnant women compared to the samples of healthy controls, which is supported by previous literature (22, 23). In a healthy, iodine-sufficient pregnant population, this is an observation considered normal physiology and not the result of a technical artifact of the measurement method. Although there has been no definite explanation for why serum fT4 concentration is decreased during pregnancy, several mechanisms are suggested to underlie this observation. HCG increases estrogen concentrations during pregnancy and consequently, estrogen increases TBG concentrations causing more T4 to bind to TBG which might lead to short-term lower freely circulating T4 (24). On the other hand, albumin concentrations decrease during pregnancy because of a dilution effect caused by an increased total blood volume (25). Although the changes in binding protein concentration will alter the total amount of bound T4, the fT4 concentration is dependent upon feedback from the pituitary by thyroid-stimulating hormone (TSH). A short-term inappropriately decreased fT4 will be sensed by the pituitary and lead to an increased TSH and stimulation of the thyroid gland to produce T4 which will ultimately result in an fT4 suitable for normal physiology during pregnancy. As another explanation, it is suggested that hemodynamic changes (e.g. increase of total blood volume) during pregnancy may lead to a lower fT4 concentration because of a dilution effect. In addition, increased type 3 deiodinase activity in the placenta is suggested to cause increased consumption of maternal thyroid hormones as well (26). On the other hand, it has also been proposed that aforementioned factors are not the cause of decreased fT4 concentrations but that the lower fT4 concentrations during pregnancy resemble the changes seen during non-thyroidal illness (NTI) (27). The observed decrease in fT4 concentration during pregnancy is, however, not worrisome because of an adaption of thyroid hormone-responsive cells by increasing the capacity of nuclear receptors (28). Such adaption can compensate for the lower fT4 concentrations and indicates altered thyroid homeostasis during pregnancy.

Secondly, we showed that all tested IAs measured lower fT4 concentrations compared to the LC-MS/MS in healthy controls, indicating a systematic bias which has been described previously (19). Indeed the International Federation of Clinical Chemistry working group for Standardization of Thyroid Function Tests acknowledged the need for standardization of the fT4 assay. Standardization will improve the comparability of the fT4 assays enabling worldwide generalized reference intervals, will improve interpretation and will prevent miscommunication regarding fT4 results. However, there are other methodological quality aspects (like matrix effects in a pregnant population as demonstrated in this study) that need to be addressed in addition to implementation of standardization (29). Although less than in healthy controls, most IAs measured significantly lower fT4 concentrations in serum of pregnant women compared to the LC-MS/MS as well. Atellica and Cobas IAs, however, did not measure significantly lower. As will be discussed in detail below, the Atellica and Cobas IAs showed the largest positive bias in serum of pregnant women. This explains the fT4 concentration measured using these IAs was (falsely) higher and reached a comparable level in pregnant women to the concentrations measured by the LC-MS/MS. This example shows the complexity of the process of fT4 standardization for all types of patients and their specific matrices.

Correction of a systematic bias by standardization may improve the comparability of the fT4 assays in healthy controls yet will not directly lead to accurate IA measurements in pregnant women. Furthermore, even though fT4 concentrations measured using both IAs and the LC-MS/MS are lower during pregnancy, the degree of this decrease is method-dependent in the immunoassays which can, thus, be attributed to methodical differences. Our study showed that in all tested IAs, fT4 concentrations in samples of pregnant women deviated positively from the LC-MS/MS method (+7.2 to +28.7%), meaning that all IAs overestimate the thyroid hormone status in pregnant women. The degree to which IAs differ is consistent with literature (12, 30, 31). Nonetheless, our study is unique in showing these results with multiple IAs currently and frequently used in clinical laboratories set against a (candidate) reference method. Although TSH within the reference interval will most likely not be followed by the measurement of fT4, the overestimation of fT4 in pregnant women could especially have consequences if TSH is deviant and fT4 is around the lower or upper limits of the reference interval if these reference intervals are not adjusted correctly for pregnancy. To illustrate, fT4 concentrations in pregnant women around the lower limit but within the reference interval of healthy controls measured using an IA may be below the reference limit when measured using LC-MS/MS. Even if standardization of the IA toward the LC-MS/MS for healthy controls has been performed, this does not solve the deviation between pregnant women and healthy controls and may lead to under diagnosis of maternal hypothyroidism. In most cases, TSH is measured first and will be increased in case of (subclinical) hypothyroidism, even though fT4 may not be lowered (yet). The distinction between subclinical hypothyroidism and overt hypothyroidism can easily be missed if fT4 is erroneously within the reference intervals. Subclinical hypothyroidism in pregnancy is treated with levothyroxine only under specific conditions, whereas it is strongly recommended to treat overt hypothyroidism in pregnancy with levothyroxine (32). This clear difference in treatment strategy indicates the relevance of our findings. Our study was not designed to determine the extent to which this phenomenon causes problems in clinical practice, so more research is needed. At the upper limits of the reference interval, an overestimation of thyroid function could have opposite consequences; pregnant women could be suspected of hyperthyroidism due to erroneously increased fT4 concentrations. However, the latter will probably have less impact because TSH is most likely not deviant in this case.

Previous literature suggested that alterations in TBG and albumin could cause fT4 assay inaccuracies (11, 12, 33, 34, 35), whereas it is most likely that the fT4 LC-MS/MS reference method does not show protein-binding-dependent aberrations due to strict adherence to the defined conventions (20) (Jansen *et al.* in preparation). Our results confirmed that TBG concentrations were significantly higher and albumin concentrations were significantly lower in samples of pregnant women compared to healthy controls (36, 37). However, the influence of albumin on fT4 assay accuracy during pregnancy has only been investigated using RIAs which cannot directly be extrapolated to automated IAs (13, 14). The influence of altered concentrations of TBG on IA measurements has been better established, although its influence during pregnancy is less evident (12, 33). Combined results of ours and previous studies showed that TBG and albumin concentrations may play a role in the fT4 IA bias in pregnant women, although a causal correlation cannot be confirmed (11, 38). Since TBG concentrations may play a role in fT4 IA inaccuracies, this might have consequences in other specific groups characterized by increased TBG concentrations, such as women using oral contraceptives or using estrogens after menopause, as well. Clearly, more research is needed to investigate other specific groups.

Our results indicate problems in the accuracy of measuring fT4 concentrations in pregnant women using IAs. Supported by both previous studies and the current study, measurement of fT4 using an LC-MS/MS reference method is thought to measure fT4 more accurately and could even be recommended for clinical use (22, 39, 40). However, the LC-MS/MS method, preceded by equilibrium dialysis, for measuring fT4 is technically demanding and time- and labor-intensive, making this method for routine clinical laboratories not useful as an alternative measurement method for the measurement of fT4 in samples from pregnant women. Since a proper solution to overcome the aforementioned issues has not been found yet, physicians and laboratory specialists should be aware that matrix effects can cause an fT4 IA bias to avoid drawing the wrong conclusions about thyroid function in pregnant women. Looking for an enduring solution, we encourage manufacturers to improve their fT4 IAs, making the assays more suitable for specific groups with, for example, deviated concentrations of binding globulins. Until then, assay-specific reference intervals for pregnant women, ideally per trimester, are necessary. Reference intervals in pregnant women for different trimesters were established for several immunoassays of Roche, Abbott, Beckman Coulter and Siemens and an ultrafiltration LC-MS/MS method as well (41, 42, 43, 44, 45, 46, 47, 48, 49, 50, 51). Studies evaluating reference intervals using the identical IA demonstrated a high consistency with comparable results and a maximal difference of 1–2 pmol/L at the lower or upper reference range (46, 47, 48). However, different fT4 IAs have varying techniques and reagent composition in measuring fT4 concentrations, meaning pregnancy does not influence all different IAs to the same extent as was shown in our study. Therefore, reference intervals for pregnant women need to be established for every IA separately and cannot be used from or recalculated based on other IAs, which has also been highlighted previously by Bliddal *et al.* (52) and Okosieme *et al.* (53). Furthermore, patient populations highly differ between countries or regions which could lead to altered reference ranges of thyroid hormone parameters (54). Our study emphasized that every laboratory should determine their own fT4 reference intervals for their pregnant population and IA of use or implement fT4 reference intervals from other laboratories using the same immunoassay and based on a comparable population of pregnant women.

In our study, pregnant women were not selected based on their trimester, so we could not differentiate between inaccuracies in different trimesters during pregnancy. This could be considered a limitation of this study; however, changes in serum matrix of pregnant women are present throughout the whole pregnancy. This study aimed to provide insight into the extent of IA difficulties during pregnancy compared to a candidate conventional reference method, meaning specification per trimester was not essential. Both male and female healthy controls were included in this study. Even though this may seem a limitation, the design of our study did not require perfectly matched groups to demonstrate the accuracy of fT4 IAs in pregnant women. Moreover, time of blood withdrawal and fasting state varied between pregnant women and healthy controls. Although this may be a limitation, literature showed that these circumstances do not influence fT4 concentrations within individuals to a large degree (55). Furthermore, other factors that might influence fT4 concentrations (e.g. smoking status, BMI, ethnicity) were not taken into account in this study (54, 56, 57, 58). Nonetheless, fT4 concentrations in pregnant women were comparable to previous studies, indicating pregnancy was the main component causing altered fT4 concentrations. In this study, we did not include participants with thyroid diseases. In general, literature showed that IAs have more difficulties in measuring fT4 concentrations far above the upper and lower limit of the reference interval (59, 60), meaning fT4 IAs could even be less accurate in pregnant women displaying severe thyroid dysfunction. Therefore, future research should focus on the accuracy of fT4 IAs in this specific group as well. The inclusion of a candidate-reference method and several commonly used IAs are strengths of this study, as the results are highly accurate and can be generalized directly to most clinical laboratories.

In conclusion, our study showed that immunoassays measure falsely high fT4 concentrations in pregnant women compared to healthy controls, leading to an overestimation of thyroid hormone status and providing insight into the extent of immunoassay difficulties during pregnancy by using an fT4 candidate-reference method. Assay-specific fT4 reference intervals for pregnant women should be established to ascertain reliable interpretation, together with an (overall) improvement of the assay by the manufacturers.

## Declaration of interest

The authors declare that there is no conflict of interest that could be perceived as prejudicing the impartiality of the research reported.

## Funding

This work did not receive any specific grant from any funding agency in the public, commercial or not-for-profit sector.

## Author contribution statement

H J collected and analyzed the data and wrote the manuscript. R P provided support in collecting the data and reviewed and edited the manuscript. A E H, H H, and J H organized the measurements and reviewed and edited the manuscript. A B conceived the study and reviewed and edited the manuscript. A C H conceived the study, organized the measurements and reviewed and edited the manuscript.
